# Adenocarcinoma and squamous cell carcinoma in the same lobe of the lung with adenocarcinoma metastasis in the lymph nodes: a case report and literature review

**DOI:** 10.3389/fonc.2025.1618619

**Published:** 2025-07-17

**Authors:** Chun-Sen Li, Yun-Xuan Zou, Chun-Yan Wang, Guo-Dong Xu, Mai-Qing Yang

**Affiliations:** ^1^ Department of Pathology, Weifang People’s Hospital (First Affiliated Hospital of Shandong Second Medical University), Weifang, Shandong, China; ^2^ Department of Anesthesiology, Weifang People’s Hospital (First Affiliated Hospital of Shandong Second Medical University), Weifang, Shandong, China; ^3^ Department of Family Planning, Weifang People’s Hospital (First Affiliated Hospital of Shandong Second Medical University), Weifang, Shandong, China

**Keywords:** adenocarcinoma, squamous cell carcinoma, lung, immunohistochemical, diagnosis

## Abstract

Adenocarcinoma and squamous cell carcinoma (SCC) of the same lobe of the lung is relatively rare. A 57-year-old man was admitted to Weifang People’s Hospital (Weifang, China) with blood streaks in sputum and had masses in the lung. Based on clinical, imaging, and pathological findings, the patient was diagnosed with primary adenocarcinoma and SCC in the same lobe of the right lung, with adenocarcinoma metastasis to the lymph nodes. Few reports have described the synchronous occurrence of adenocarcinoma and SCC in the same lobe. Thoracoscopic resection of the lower lobes of the right lung and mediastinal lymph node dissection were performed. Surgical resection and postoperative chemotherapy have superior effects. The misdiagnosis of this tumor as other types of tumor must be prevented. Immunohistochemical features can be useful for the diagnosis of primary adenocarcinoma and SCC.

## 1 Introduction

Synchronous multiple primary lung cancer (sMPLC) is a special type of lung cancer characterized by the simultaneous identification of ≥ 2 primary tumors in the ipsilateral or contralateral lungs (1). The incidence of sMPLC ranges from 0.2–20% in lung cancer (1–4). Most of these tumors have the same histological type (4, 5). Owing to the increase in detection rates, the proportion of sMPLC in lung cancer and five‐year survival rate are increasing, whereas postoperative mortality is decreasing gradually (1, 6–8). The clinical diagnosis of MPLC is predominantly based on the Martini-Melamed diagnostic criteria, which primarily rely on tumor location, histologic features, presence or absence of carcinoma *in situ*, and other characteristics (9). The American College of Chest Physicians (ACCP) and the International Association for the Study of Lung Cancer (IASLC) proposed updated diagnostic criteria (10). The 2016 IASLC criterion provided a more detailed description of MPLC diagnostic criteria, incorporating the Comprehensive Histologic Assessment (CHA) process (11). This paper reports an unusual case of synchronous adenocarcinoma and squamous cell carcinoma (SCC) in the same lobe of the lung with adenocarcinoma metastasis to the lymph nodes to strengthen our understanding of this disease. Knowingly, including the present case, only six patients with primary synchronous occurrence of adenocarcinoma and SCC in the same lobe have been reported (4, 12–14).

## 2 Case presentation

### 2.1 Clinical history

**Figure 1:**
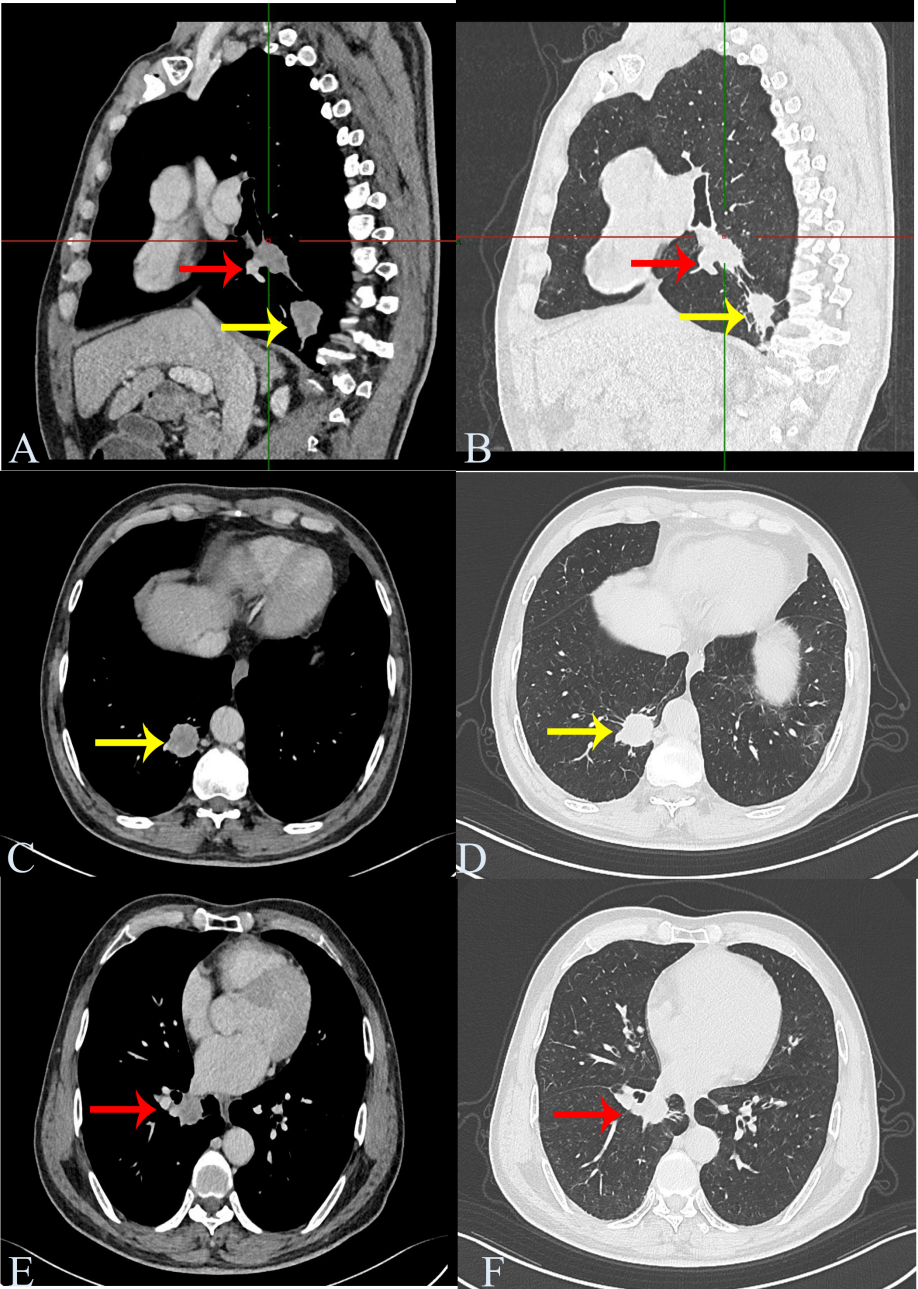
Chest computed tomography. Computed tomography shows a nodule in the posterior basal segment of the lower lobe of the right lung, approximately 3.1 cm × 2.6 cm in size (nodule 1, yellow arrow). Another nodule is seen in the medial basal segment of the lower lobe of the right lung, approximately 2.6 cm × 2.3 cm in size (nodule 2, red arrow). **(A, B)** Sagittal screenshot. **(C, D)** Axis position screenshot of nodule 1. **(E, F)** Axis position screenshot of nodule 2.

A 57-year-old man was admitted to our hospital for further treatment and presented with blood streaks in sputum for > 50 days. With many years smoking history of smoking approximately 40 cigarettes daily. The patient had no additional illness and was previously in good health. Personal history, family histories, medication history, social history, and allergy history were negative. Pulmonary function test is normal. Computed tomography (CT) evaluation showed a nodule in the posterior basal segment of the lower lobe of the right lung, approximately 3.1 cm × 2.6 cm in size. Another nodule was observed in the medial basal segment of the lower lobe of the right lung, approximately 2.6 cm × 2.3 cm in size. The mediastinum was centered and enlarged lymph node shadows were visible. No pleural effusion or thickening was observed on either side of the pleura (**Figure 1**). Blood SSC antigen (SCCA) (2.97 ng/mL) was slightly higher than normal (0–2.5 ng/mL). Carcinoembryonic antigen (CEA) (1.33 ng/mL [0–4.5 ng/mL]) and neuron-specific enolase (NSE) (13.23 ng/mL [0–16.5 ng/mL]) were normal. The preoperative diagnosis was right lower-lobe lung cancer. Thoracoscopic resection of the lower lobe of the right lung was performed. Perioperatively, resected lung specimens were collected to prepare frozen sections for pathological evaluation. The diagnosis was “two places of non-small cell lung cancer (NSCLC), depending on routine paraffin sections and immunohistochemical identification and classification after the operation.” Subsequently, extended dissection of the mediastinal lymph nodes was performed.

### 2.2 Immunohistochemical staining

The specimens were fixed with 10% neutral-buffered formalin, embedded in paraffin blocks, and cut into 4 μm thick sections. The sections were stained with hematoxylin and eosin (HE) for histological assessment. Tumor sections were immunostained with ready-to-use primary antibodies against cytokeratin 7 (CK7, Catalog Number: MAB-0828), TTF-1 (thyroid transcription factor-1, Catalog Number: MAB-0677), napsin A (Catalog Number: MAB-0704), CK5/6 (Catalog Number: RMA-1144), P40 (Catalog Number: RMA-1006), and Ki-67 (Catalog Number: MAB-0672, all were from Maixin Biotech Co., Ltd., Fuzhou, China). Immunohistochemistry was performed using EnVision. Positive and negative controls were used as appropriate. All results were diagnosed independently by two pathologists.

### 2.3 Pathological diagnosis and follow-up history

**Figure 2:**
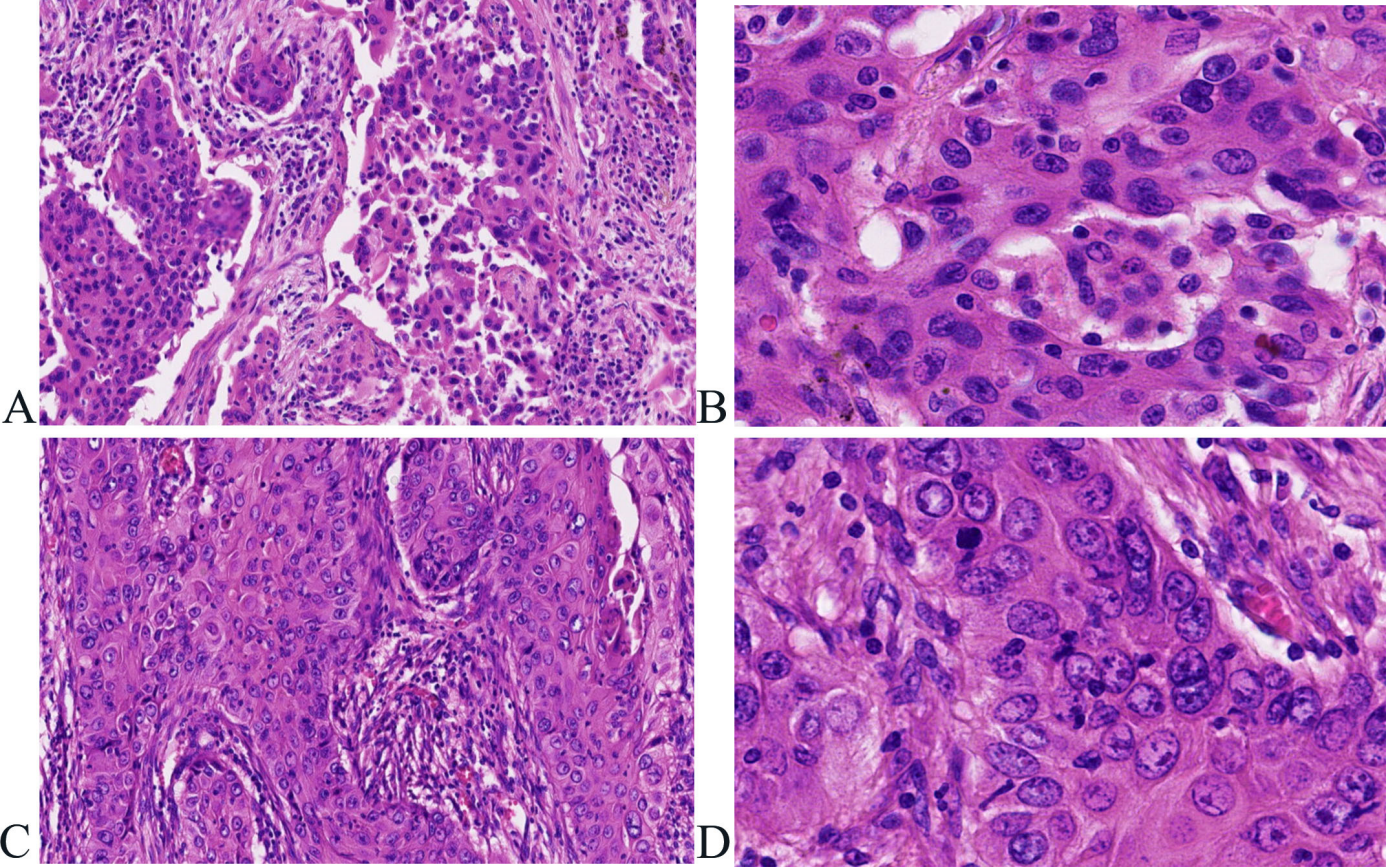
Histological features associated with adenocarcinoma and squamous cell carcinoma in the same lobe of the lung. **(A, B)** Tumor cells in “nodule 1” mostly grow in complex glandular pattern, solid **(A)**), hematoxylin and eosin, ×100), with large nucleoli and displaced nucleoli **(B)**), hematoxylin and eosin, ×400). **(C, D)** Tumor cells form irregular nests and strands of tumor cells separated by various amounts of fibrous stroma **(C)**), hematoxylin and eosin, ×100) with cytoplasmic keratosis and no keratin pearl **(D)**), hematoxylin and eosin, ×400).

**Figure 3:**
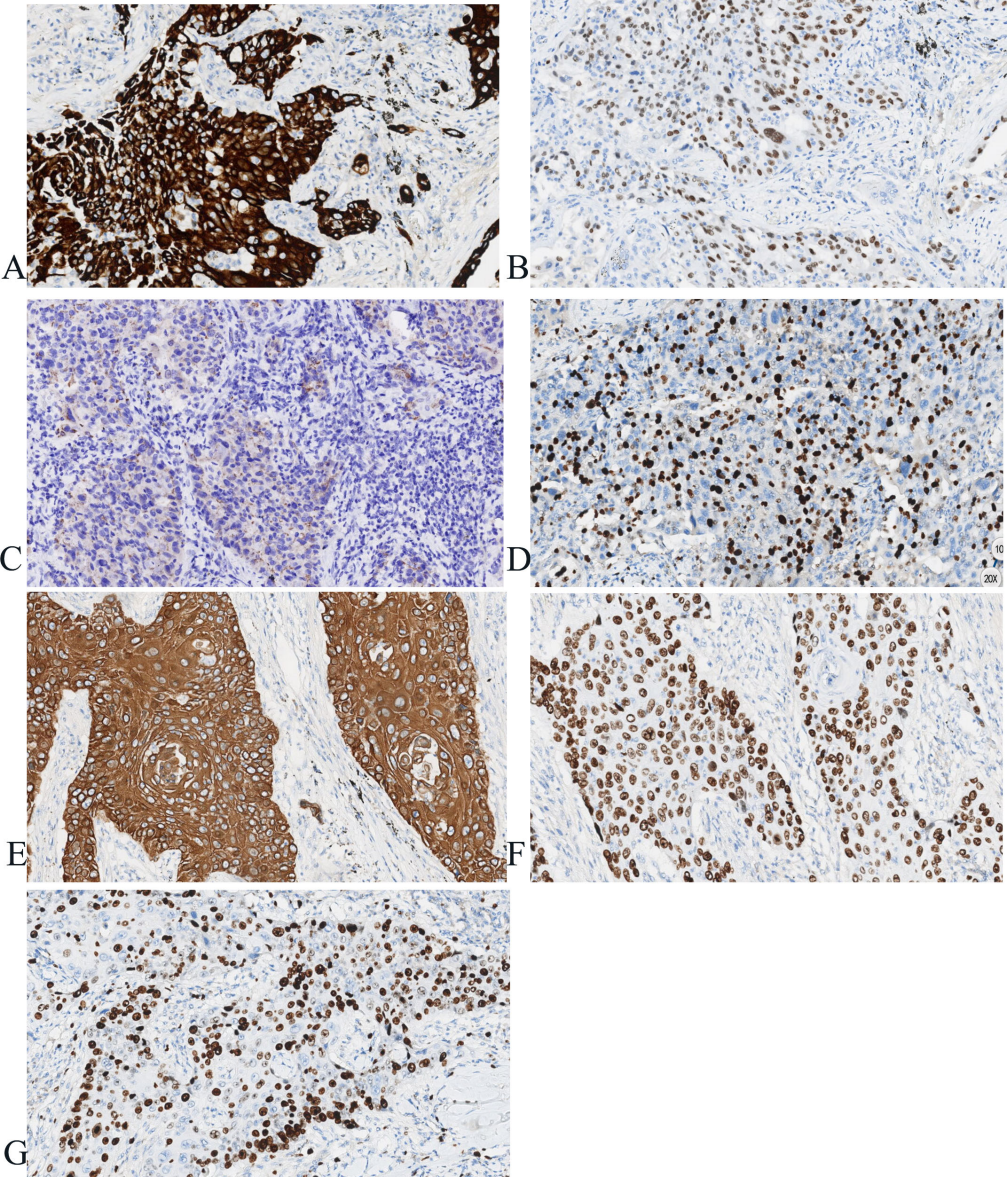
Immunohistochemistry of adenocarcinoma and squamous cell carcinoma in the same lobe of the lung. **(A–D)** Immunohistochemically, the tumor cells show positive expression of CK7, TTF-1, napsin A, Ki-67 (approximately 35% in hot spots) in nodule 1 (×100). **(E–G)** Immunohistochemically, the tumor cells showed positive expression of CK5/6, P40, Ki-67 (approximately 45% in hot spots) in nodule 2 (×100).

Postoperatively, tumor specimens were embedded in paraffin blocks and examined. A lobe of the lung, 14 cm×11 cm×3.5 cm in volume, was resected 4 cm away from the bronchial end close to the lung capsule, nodule 1, 3.5 cm×2.2 cm×2 cm in volume, was seen with a grayish, hard section surface and unclear boundary. Nodule 2, 2.8 cm× 2.8 cm×1.7 cm in volume, was seen 0.5 cm away from the bronchial end and 1.5 cm from the lung capsule, with gray-white, hard section surface and unclear boundary. Microscopically, the tumor cells in “nodule 1” mostly grew in cribriform and solid patchy forms with large and displaced nucleoli (**Figure 2**). Immunohistochemically, the tumor cells showed positive expression of CK7, TTF-1, napsin A, and Ki-67 (approximately 35% in hot spots), and were negative for CK5/6 and P40 (**Figure 3**). “Nodule 1” was revealed poor differentiation invasive adenocarcinoma. The proportions of tumor growth patterns in the complex glandular pattern, solid, and acinar without pleural invasion were 55%, 35%, and 10%, respectively. Histological examination of “nodule 2” revealed that the carcinomas formed irregular nests and strands of tumor cells separated by various amounts of fibrous stroma with cytoplasmic keratosis and no keratin pearls. Mitotic figures and necrosis were common (**Figure 2**). Immunohistochemically, the tumor cells were positive for CK5/6, P40, and Ki-67 (approximately 45% in hot spots) and negative for TTF-1 and napsin A (**Figure 3**). “Nodule 2” was a moderately differentiated SCC. Adenocarcinoma metastases were observed in the lymph nodes (8/15, 1/1, 0/2, 0/3, and 0/2 in groups 7, 10, 2, 4, and 11, respectively). Immunohistochemically, the metastatic tumor cells were positive for TTF-1 and negative for P40.

The final diagnosis was lung adenocarcinoma combined with SCC and T2N2M0 according to the ninth edition of the tumor node metastasis (TNM) staging system and successfully discharged from the hospital after five days. Based on the patient’s wishes, only immunohistochemistry was performed; sequencing and other related examinations were incomplete. The patient received systemic chemotherapy with paclitaxel and cisplatin postoperatively and recovered well. Follow-up at three months before the deadline for submission did not reveal any evidence of recurrence or other metastatic diseases.

## 3 Discussion

Based on the clinical information and immunohistochemical results, the pathological diagnoses were primary adenocarcinoma and SCC of the lower lobe of the right lung. This case study presents a complex and diagnostically challenging scenario. Such a combination is relatively uncommon compared with solitary lung malignancies and requires a comprehensive understanding.

MPLC are cases with > 1 primary cancer occurring simultaneously or sequentially in the lungs (4). For sMPLC, tumors should be physically separated and histologically identical or different (15). The prevalence of low-dose CT lung cancer screening programs has boosted lung cancer diagnosis (16), and MPLC is becoming a common phenomenon in clinical practice (8, 15). Since Martini first proposed the diagnostic criteria (9), MPLC has gained significant attention.

Most MPLC have the same histological type (4, 5). Few reports have described the synchronous occurrence of adenocarcinoma and SCC in the same lobe. The first such case was reported by Kitamura et al., 1991 (12). Knowingly, including the present case, only six patients with primary synchronous occurrence of adenocarcinoma and SCC in the same lobe have been reported (4, 12–14). The clinicopathological features of the patients are summarized in **Table 1**. Six male patients (age; 57–77 years, mean age; 66.5 years). Most had a smoking history (four smokers, one former smoker, and one unknown). Only one of our patients had lymph node metastasis. All the patients underwent surgical resection. Three cases were located in the left lobe and three cases were located in the right lobe, and except for our case, the remaining five cases are all located in the upper lobe. The location of lung cancer in the upper or lower lobe may have some impact on prognosis, but it is not an independent decisive factor. Yngvar Nilssen et al. found that the right upper lobe comprised 31.2% of the tumors and 17.6% of the lung volume. The relative proportion of 1.77 was higher than in the other lobes (17). Hyun Woo Lee et al. found patients with lower lobe cancer showed a higher all-cause mortality rate than those with non-lower lobe cancer (18). Lymph node involvement in any primary tumor may upstage the overall disease and lead to a poor prognosis. Unfortunately, none of the other five cases had a complete follow-up history, and our patient was only followed up for three months before the deadline for submission, and we will continue to follow up.

**Table 1:** Summary of primary adenocarcinoma and squamous cell carcinoma in the same lung features.

No.	Year	Sex	Age	Smoker	Size(cm)	Site	Pathological diagnosis	Metastasis	Therapy	Outcome
1 (12)	1991	M	70	Yes	①1.7×1.7 ②1.8×1.7	right upper lobe	①Adec ②SCC	No	surgical resection	unknown
2 (13)	2017	M	58	Yes	①3.7×2.0 ②4.5×3.2	upper lobe of the left lung.	①Adec ②SCC	No	Left upper lobectomy with lymph node dissection	unknown
3 (14)	2020	M	72	Yes	①1.6 ②1.6	right upper lobe and middle lobe	①Adec ②SCC	No	surgical resection	unknown
4 (14)	2020	M	77	Former smoker	①1.3 ②1.6	left upper lobe	①Adec ②SCC	No	surgical resection	unknown
5 (4)	2024	M	65	unknown	①1.0 ②3.9×3.0	left upper lobe	①Adec ②SCC	No	lobectomy of the left upper lung and mediastinal lymph nodes, adjuvant treatment	unknown
6 (present case)	2025	M	57	Yes	①3.1×2.6 ②2.6×2.3	lower lobe of the right lung	①Adec ②SCC	Yes	resection of lower lobe of the right lung, dissection of mediastinal lymph nodes, and chemotherapy	Alive free of disease, 3mo

M, male; mo, month; Adec, adenocarcinoma; SCC, squamous cell carcinoma.

The incidence of MPLC remains controversial because of varying diagnostic criteria and overlapping intrapulmonary metastases. Studies suggest that MPLC accounts for 0.2–20% of lung cancer (1–4), with an increasing trend attributed to improved diagnostic techniques and increased survival of patients with early stage lung cancer (8, 15, 16). The pathogenesis of MPLC involves complex interactions between genetic predispositions and environmental exposure. Smoking remains a major risk factor (19–21). Chronic exposure to carcinogens creates a “field” of genetically damaged epithelial cells, increasing the risk of multiple independent primaries. This is supported by studies showing shared somatic mutations in adjacent normal lung tissue of smokers with MPLC (22). However, the genetic background of patients with MPLCs remains unclear (23). Molecular studies have indicated that MPLC tumors often exhibit distinct genetic profiles, such as epidermal growth factor receptor (EGFR), Kirsten rat sarcoma viral oncogene (KRAS), Erb-B2 Receptor Tyrosine Kinase 2 (ERBB2) tumor protein 53 (TP53), which support their independent clonal origin (24–26). Genomic analyses have shown that multiple primary lesions are much more heterogeneous, unlike metastatic lesions (25). Chronic inflammation may promote clonal expansion of mutated cells and suppress antitumor immunity in MPLC (27). Unfortunately, because the patient refused to have genetic detection, there were no results of genetic detection in this case.

The management of MPLC requires a multidisciplinary approach that prioritizes curative intent for resectable lesions. Surgical resection remains the cornerstone of treatment (28, 29). MPLC with EGFR, anaplastic lymphoma kinase, or ROS proto-oncogene 1, receptor tyrosine kinase alterations in individual lesions may benefit from lesion-specific targeted therapy (26). MPLC with high PD-L1 expression (xpres or mismatch repair deficiency may respond to immune checkpoint inhibitors (27).

A key challenge is differentiating MPLC from metastases to avoid overtreatment (30). The clinical diagnosis of MPLC is predominantly based on the Martini-Melamed diagnostic criteria, which primarily rely on tumor location, histologic features, presence or absence of carcinoma *in situ*, and other characteristics (9). The American College of Chest Physicians (ACCP) and the International Association for the Study of Lung Cancer (IASLC) proposed updated diagnostic criteria (10). The 2016 IASLC criterion provided a more detailed description of MPLC diagnostic criteria, incorporating the Comprehensive Histologic Assessment (CHA) process (11). Accurate diagnosis of MPLC is based on histologic type and onset interval and does not incorporate genetic analysis (15, 23). Microscopic morphology and immunohistochemistry are helpful for differential diagnosis. SCC usually exhibits pronounced keratinization and intercellular bridges and is diffusely positive for P63 and P40; adenocarcinoma is positive for TTF-1, napsin-A and neuroendocrine tumors are positive for CD56, synaptophysin, and chromogranin (31, 32).

Studies have indicated that patients with early stage MPLC have better survival outcomes than those with metastatic disease (15, 28). Poor prognostic factors include advanced tumor stage and lymph node involvement (33, 34). Chest CT may predict unexpected recurrence and metastasis after radical surgery for MPLC (35). In the future single-cell RNA sequencing may identify distinct immune cell profiles in MPLC (36). Liquid biopsy for clonality assessment may distinguish MPLC from metastasis (37).

Overall, we report a rare case of primary synchronous adenocarcinoma and SCC in the same lobe of the lung with adenocarcinoma metastasis to the lymph nodes. Complete surgical resection was the treatment of choice. Careful assessment of the histological features and immunohistochemistry enables an efficient diagnosis.

## Data Availability

The original contributions presented in the study are included in the article/supplementary material. Further inquiries can be directed to the corresponding author.

## References

[1] Tie H Luo J Shi R Li Z Chen D Wu Q . Characteristics and prognosis of synchronous multiple primary lung cancer after surgical treatment: A systematic review and meta-analysis of current evidence. Cancer Med. (2021) 10:507–20. doi: 10.1002/cam4.3614, PMID: 33300681 PMC7877344

[2] Li M Wan Y Zhang L Zhou LN Shi Z Zhang R . Synchronous multiple lung cancers presenting as multifocal pure ground glass nodules: are whole-body positron emission tomography/computed tomography and brain enhanced magnetic resonance imaging necessary? Transl Lung Cancer Res. (2019) 8:649–57. doi: 10.21037/tlcr.2019.09.10, PMID: 31737500 PMC6835114

[3] Ferguson MK DeMeester TR DesLauriers J Little AG Piraux M Golomb H . Diagnosis and management of synchronous lung cancers. J Thorac Cardiovasc Surg. (1985) 89:378–85. doi: 10.1016/S0022-5223(19)38787-2 3974273

[4] Zhang T He R Xiao Y Geng Q . Primary squamous cell carcinoma and adenocarcinoma simultaneously occurring in the same lung lobe: A case report and literature review. Front Oncol. (2024) 14:1402297. doi: 10.3389/fonc.2024.1402297, PMID: 38800406 PMC11116623

[5] Romaszko AM Doboszyńska A . Multiple primary lung cancer: A literature review. Adv Clin Exp medicine: Off Organ Wroclaw Med Univ. (2018) 27:725–30. doi: 10.17219/acem/68631, PMID: 29790681

[6] Zhang Z Gao S Mao Y Mu J Xue Q Feng X . Surgical outcomes of synchronous multiple primary non-small cell lung cancers. Sci Rep. (2016) 6:23252. doi: 10.1038/srep23252, PMID: 27254665 PMC4890551

[7] Voltolini L Rapicetta C Luzzi L Ghiribelli C Paladini P Granato F . Surgical treatment of synchronous multiple lung cancer located in a different lobe or lung: high survival in node-negative subgroup. Eur J Cardiothorac Surg. (2010) 37:1198–204. doi: 10.1016/j.ejcts.2009.11.025, PMID: 20022516

[8] Wang Z Zhang Q Wang C Herth FJF Guo Z Zhang X . Multiple primary lung cancer: updates and perspectives. Int J Cancer. (2024) 155:785–99. doi: 10.1002/ijc.34994, PMID: 38783577

[9] Martini N Melamed MR . Multiple primary lung cancers. J Thorac Cardiovasc Surg. (1975) 70:606–12. doi: 10.1016/S0022-5223(19)40289-4 170482

[10] Shen KR Meyers BF Larner JM Jones DR . Special treatment issues in lung cancer: accp evidence-based clinical practice guidelines (2nd edition). Chest. (2007) 132:290s–305s. doi: 10.1378/chest.07-1382, PMID: 17873175

[11] Detterbeck FC Nicholson AG Franklin WA Marom EM Travis WD Girard N . The iaslc lung cancer staging project: summary of proposals for revisions of the classification of lung cancers with multiple pulmonary sites of involvement in the forthcoming eighth edition of the tnm classification. J Thorac oncology: Off Publ Int Assoc Study Lung Cancer. (2016) 11:639–50. doi: 10.1016/j.jtho.2016.01.024, PMID: 26940528

[12] Kitamura K Mitsudomi T Ishida T Kaneko S Sugimachi K . Adenocarcinoma and squamous cell carcinoma in the same lobe of the lung. A case report. Respiration. (1991) 58:226–8. doi: 10.1159/000195933, PMID: 1745862

[13] Bacalja J Tomasović Lončarić Č Kukulj S Nikolić I . The case of synchronous occurrence of primary adenocarcinoma and squamous cell carcinoma in the same lobe of the lung. Acta Clin Belg. (2017) 72:289–92. doi: 10.1080/17843286.2016.1237697, PMID: 27667399

[14] Wu L Kang P Tao S Zhao Z Chen L Xiao Y . Genomic profiles and transcriptomic microenvironments in 2 patients with synchronous lung adenocarcinoma and lung squamous cell carcinoma: A case report. BMC Med Genomics. (2020) 13:15. doi: 10.1186/s12920-020-0663-8, PMID: 32005243 PMC6995067

[15] Tian H Bai G Yang Z Chen P Xu J Liu T . Multiple primary lung cancer: updates of clinical management and genomic features. Front Oncol. (2023) 13:1034752. doi: 10.3389/fonc.2023.1034752, PMID: 36910635 PMC9993658

[16] Sands J Tammemägi MC Couraud S Baldwin DR Borondy-Kitts A Yankelevitz D . Lung screening benefits and challenges: A review of the data and outline for implementation. J Thorac oncology: Off Publ Int Assoc Study Lung Cancer. (2021) 16:37–53. doi: 10.1016/j.jtho.2020.10.127, PMID: 33188913

[17] Nilssen Y Brustugun OT Fjellbirkeland L Helland Å Møller B Wahl SGF . Distribution and characteristics of Malignant tumours by lung lobe. BMC pulmonary Med. (2024) 24:106. doi: 10.1186/s12890-024-02918-w, PMID: 38439038 PMC10910834

[18] Lee HW Park YS Park S Lee CH . Poor prognosis of nsclc located in lower lobe is partly mediated by lower frequency of egfr mutations. Sci Rep. (2020) 10:14933. doi: 10.1038/s41598-020-71996-7, PMID: 32913267 PMC7483476

[19] Herbst RS Morgensztern D Boshoff C . The biology and management of non-small cell lung cancer. Nature. (2018) 553:446–54. doi: 10.1038/nature25183, PMID: 29364287

[20] Ni CH Wang MT Lu YQ Zheng W Chen C Zheng B . Association between a family history of cancer and multiple primary lung cancer risks: A population-based analysis from China. BMC pulmonary Med. (2023) 23:415. doi: 10.1186/s12890-023-02676-1, PMID: 37907909 PMC10619319

[21] Sugimura H Watanabe S Tsugane S Morinaga S Yoneyama T . Case-control study on histologically determined multiple primary lung cancer. J Natl Cancer Institute. (1987) 79:435–41., PMID: 3476786

[22] Chandwani R Brokamp C Salfity H Starnes SL Van Haren RM . Impact of environmental exposures on lung cancer in patients who never smoked. World J Surg. (2023) 47:2578–86. doi: 10.1007/s00268-023-07085-3, PMID: 37402836

[23] Wu CT Lin MW Hsieh MS Kuo SW Chang YL . New aspects of the clinicopathology and genetic profile of metachronous multiple lung cancers. Ann Surg. (2014) 259:1018–24. doi: 10.1097/sla.0000000000000385, PMID: 24368645

[24] van Dekken H Wink JC Vissers KJ van Marion R Franken PF Hoogmans MM . Genomic analysis of a case of multifocal adenocarcinoma in ulcerative colitis. Virchows Archiv: an Int J Pathol. (2006) 449:716–21. doi: 10.1007/s00428-006-0312-4, PMID: 17091253

[25] Asmar R Sonett JR Singh G Mansukhani MM Borczuk AC . Use of oncogenic driver mutations in staging of multiple primary lung carcinomas: A single-center experience. J Thorac oncology: Off Publ Int Assoc Study Lung Cancer. (2017) 12:1524–35. doi: 10.1016/j.jtho.2017.06.012, PMID: 28647671

[26] Liang Z Zeng G Wan W Deng B Chen C Li F . The unique genetic mutation characteristics based on large panel next-generation sequencing (Ngs) detection in multiple primary lung cancers (Mplc) patients. Discov Med. (2023) 35:131–43. doi: 10.24976/Discov.Med.202335175.14, PMID: 37188510

[27] Yang Z Zhou B Guo W Peng Y Tian H Xu J . Genomic characteristics and immune landscape of super multiple primary lung cancer. EBioMedicine. (2024) 101:105019. doi: 10.1016/j.ebiom.2024.105019, PMID: 38364701 PMC10878856

[28] Huang Y Zhang HM Cai HR Hu JF . Advances in the diagnosis and treatment of multiple primary lung cancers. Asian J Surg. (2024) 47:2635–6. doi: 10.1016/j.asjsur.2024.03.022, PMID: 38548539

[29] Usuda J Ichinose S Ishizumi T Hayashi H Ohtani K Maehara S . Management of multiple primary lung cancer in patients with centrally located early cancer lesions. J Thorac oncology: Off Publ Int Assoc Study Lung Cancer. (2010) 5:62–8. doi: 10.1097/JTO.0b013e3181c42287, PMID: 19952800

[30] Wang Y Chen D Liu Y Shi D Duan C Li J . Multidirectional characterization of cellular composition and spatial architecture in human multiple primary lung cancers. Cell Death Dis. (2023) 14:462. doi: 10.1038/s41419-023-05992-w, PMID: 37488117 PMC10366158

[31] Xu L Chen J Zeng Y Li X Zhang Z . Differential diagnosis of multiple primary lung cancers and intra-lung metastasis of lung cancer by multiple gene detection. Chin Med J. (2022) 135:86–8. doi: 10.1097/cm9.0000000000001739, PMID: 34759216 PMC8850826

[32] Latimer KM Mott TF . Lung cancer: diagnosis, treatment principles, and screening. Am Fam Physician. (2015) 91:250–6., PMID: 25955626

[33] Wu SC Lin ZQ Xu CW Koo KS Huang OL Xie DQ . Multiple primary lung cancers. Chest. (1987) 92:892–6. doi: 10.1378/chest.92.5.892, PMID: 3665605

[34] Jiang L He J Shi X Shen J Liang W Yang C . Prognosis of synchronous and metachronous multiple primary lung cancers: systematic review and meta-analysis. Lung Cancer (Amsterdam Netherlands). (2015) 87:303–10. doi: 10.1016/j.lungcan.2014.12.013, PMID: 25617985

[35] Li S Chen G Zhang W Ma H Liu B Xu L . A novel decision tree algorithm model based on chest ct parameters to predict the risk of recurrence and metastasis in surgically resected stage I synchronous multiple primary lung cancer. Ther Adv Respir Dis. (2025) 19:17534666251325443. doi: 10.1177/17534666251325443, PMID: 40083187 PMC11907625

[36] Guo W Zhou B Bie F Huai Q Xue X Guo L . Single-cell rna sequencing analysis reveals transcriptional heterogeneity of multiple primary lung cancer. Clin Trans Med. (2023) 13:e1453. doi: 10.1002/ctm2.1453, PMID: 37846760 PMC10580343

[37] Li W Liu JB Hou LK Yu F Zhang J Wu W . Liquid biopsy in lung cancer: significance in diagnostics, prediction, and treatment monitoring. Mol Cancer. (2022) 21:25. doi: 10.1186/s12943-022-01505-z, PMID: 35057806 PMC8772097

